# Socioeconomic Status and Access to Healthcare: Interrelated Drivers for Healthy Aging

**DOI:** 10.3389/fpubh.2020.00231

**Published:** 2020-06-18

**Authors:** Darcy Jones McMaughan, Oluyomi Oloruntoba, Matthew Lee Smith

**Affiliations:** ^1^Department of Health Education and Promotion, School of Community Health Sciences, Counseling, and Counseling Psychology, Oklahoma State University, Stillwater, OK, United States; ^2^Center for Population Health and Aging, Texas A&M University, College Station, TX, United States; ^3^Department of Environmental and Occupational Health, School of Public Health, Texas A&M University, College Station, TX, United States

**Keywords:** socioeconomic status, healthcare access, access to care, healthy aging, older adults

## Abstract

The rapid growth of the global aging population has raised attention to the health and healthcare needs of older adults. The purpose of this mini-review is to: (1) elucidate the complex factors affecting the relationship between chronological age, socio-economic status (SES), access to care, and healthy aging using a SES-focused framework; (2) present examples of interventions from across the globe; and (3) offer recommendations for research-guided action to remediate the trend of older age being associated with lower SES, lack of access to care, and poorer health outcomes. Evidence supports a relationship between SES and healthcare access as well as healthcare access and health outcomes for older adults. Because financial resources are proportional to health status, efforts are needed to support older adults and the burdened healthcare system with financial resources. This can be most effective with grassroots approaches and interventions to improve SES among older adults and through data-driven policy and systems change.

## Introduction

Healthy aging, also known as successful aging (1), is defined by the World Health Organization as “the process of developing and maintaining functional ability that enables well-being in older age” (2). It encompasses the physical and mental capacities of an older adult at any given time (3) as well as the resources and supports they access and utilize. Central to the concept of healthy aging is disease and disability prevention and management; maintenance of good physical and cognitive functionality; and engagement in active lifestyles and healthful behaviors (4). Healthy aging is a primary goal of modern medicine, especially as it relates to geriatric care. Despite efforts to make healthy aging ‘the new normal (5, 6), subsets of the growing older adult population are faced with financial hardships resulting in inequities of resource distribution and disparities in health outcomes.

As our global society rapidly ages, we potentially face a future of impoverished older adults lacking access to care in already overburdened healthcare systems. In this context, healthy aging is fraught with difficulties. This mini-review: (1) discusses the relationship between chronological age, socio-economic status (SES), access to care, and healthy aging; (2) presents examples of interventions from across the globe; and (3) offers recommendations for research-guided action to remediate the trend of older age being associated with lower SES, lack of access to care, and poorer health outcomes.

**Figure 1:**
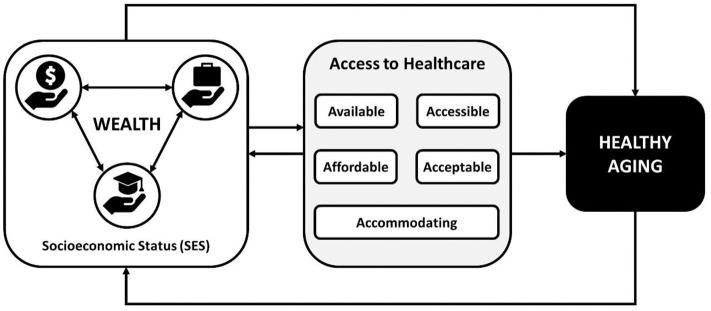
Conceptual framework for socioeconomic status and healthcare access driving healthy aging.

In the framework of this mini-review (see Figure 1), healthy aging is primarily a function of SES, with SES consisting of lifelong evolving and recursive statuses around the reflexive position of a person's financial situation, educational attainment, and employment status. A person's ability to access care is mediated by SES. Access to healthcare implies the availability of relevant and effective services and providers, physical accessibility, and affordability, how accommodating services and service providers are, and the acceptability of the services and service providers to the patient (7). Whether a population has access to healthcare is typically measured through availability (i.e., a count of providers in a defined area), utilization (i.e., rates of a target population using a certain type of healthcare service or resource), and health outcomes of the target population (8).

In addition, access to care can affect a person's SES through a downward trajectory, where (for example) poverty reduces access to healthcare, which leads to increased morbidity, which leads to increased poverty and further reductions in access to care. Context, such as rurality, neighborhood, or country has a similar relationship to healthy aging through SES. A person's context also affects access to care, as noted in areas with limited access to care due to lack of healthcare providers (like rural or remote communities). Given the relative difficulty of changing context and the extended timeframe needed to do so, it is essential to target interventions at the more immediately accessible constructs of access to care and SES through wealth and reducing financial disparities and costs of care. In a variety of contexts, lower SES is associated with reduced access to care, poorer health outcomes, and increased mortality and morbidity as individuals age (9–18). Thus, this mini-review specifically targets the relationship between wealth, access to healthcare, and healthy aging.

## Wealth, Access to Care, and Healthy Aging

Our framework emphasizes the socioeconomic gradient (or “wealth-health” gradient), which highlights the positive relationship between wealth and health. That is, as wealth increases so does health, with the converse also holding true. Lower economic status leads to poorer health, which in turn leads to a dangerous cycle of further impoverishment (19). Simply stated, there is a relationship between SES and health, with low SES associated with poorer health (20–24).

### Wealth and Healthy Aging

The “wealth-health” gradient becomes more pronounced as people age. Socioeconomic status is intimately tied to healthy aging, with greater wealth producing a greater likelihood of health among older adults (25). This association may be due to the combined effects of increased stress, trauma, allopathic load, and limited access to appropriate and timely healthcare (17, 26–28). Low SES also contributes to heavier disease burden. For example, poorer older adults experience more dental disease (29, 30) and disability (31, 32). This effect is global. In Japan, low-income older adults reached older-age with less healthy teeth intact than their higher-income counterparts (33). Poorer older adults in China and India report greater functional impairment and disability than older adults in the richest, richer, and middle-income classes (34). In Cambodia, the poorest older adults report worse health outcomes, with marginal gains in income associated with improvements in health (35). Financial instability can, in some cases, explain the poorer mortality and morbidity often found among racial and ethnic minorities compared to majority populations (36). Table 1 provides an overview of selected evidence documenting the relationship between wealth and healthy aging from different countries.

**Table 1:** Example studies documenting the relationship between wealth and healthy aging.

**References**	**Narrative Summary**	**Country**
Tashiro (33)	In Japan, low-income older adults reached older-age with less healthy teeth intact than their higher-income counterparts	Japan
Kumar (34)	Poorer older adults in China and India report greater functional impairment and disability than older adults in the richest, richer, and middle-income classes	China and India
Zimmer (35)	The poorest older adults report the worse health outcomes, with marginal gains in income associated with improvements in health	Cambodia

### Wealth and Access to Care

Socioeconomic status is tied to healthcare access among older adults, perceived or otherwise (37, 38). Variation in healthy aging based on income levels may be attributed to differential healthcare access: wealthier older adults have better access to care, and access to care (from preventative services to long-term care) may be associated with better health outcomes (21, 39, 40). Poor health-related quality of life outcomes are significantly associated with lower SES in the United States, which is possibly driven by limited healthcare access among poorer older adults (41). In a cross-sectional study of almost 50,000 non-institutionalized older adults, costs were cited as a major reason for not obtaining needed care (42). Older adults living in higher socioeconomic brackets are more likely to access preventative care and screenings, with for example, higher SES older adults experiencing a greater likelihood of having a hearing screen and using a hearing aid (43). Lower SES is associated with longer wait times in countries with centralized healthcare systems (44). Faced with rising healthcare costs, Japanese older adults report forgoing healthcare due to limited income (45). In India, financial instability is a driving factor for lower healthcare access among older adults (46). Portuguese older adults cite financial concerns (e.g., pension cuts, increased medical care fees, and increased out-of-pocket costs for medications) among the main barriers to access to care (47). In some cases, older adults with low SES are simply not offered the same care as older adults with higher incomes, resulting in income-related treatment disparities (48–50). Table 2 provides an overview of selected evidence documenting the relationship between wealth and access to care from different countries.

**Table 2:** Example studies documenting the relationship between wealth and access to care.

**References**	**Narrative Summary**	**Country**
Hugu et al. (41)	Poor health-related quality of life outcomes are significantly associated with lower SES in the	Canada/United States
Okoro et al. (42)	In a cross-sectional study of almost 50,000 non-institutionalized older adults, costs were cited as a major reason for not obtaining needed care	United States
Nieman et al. (43)	Older adults living in higher socioeconomic brackets are more likely to access preventative care and screenings, with for example, higher SES older adults experiencing a greater likelihood of having a hearing screen and using a hearing aid	United States
Murata et al. (45)	Faced with rising healthcare costs, Japanese older adults report forgoing healthcare due to limited income	Japan
Dey et al. (46)	Financial instability is a driving factor for lower healthcare access among older adults	India
Doetsch et al. (47)	Financial concerns such as pension cuts, increased medical care fees and increased out-of-pocket costs for medications are among the main barriers to access care among Portuguese older adults	Portugal

### Access to Care and Healthy Aging

**Figure 2:**
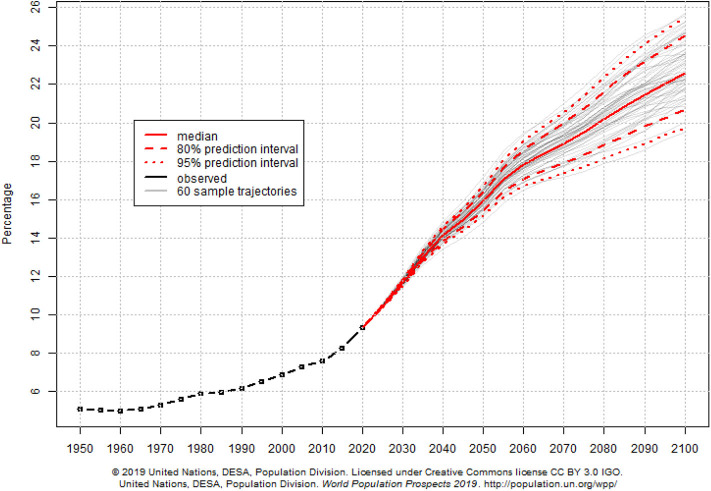
World percentage of population aged 65 years and over.

Access to healthcare is related to healthy aging. Older adults with multiple sclerosis in Canada report “effective and accessible” healthcare as a key factor in healthy aging (51). In the United States, providing health insurance coverage—a necessary conduit for access to healthcare—improves health outcomes and mortality in general and among older adults (52–55). Older adults in the United States are significantly more likely to receive clinical preventive services with access to regular sources of healthcare. (42) Similarly, for people with intellectual disabilities in the United States, access to proper screenings and preventive services facilitates healthy aging (56). In China, self-reported inadequate access to healthcare among older adults was significantly associated with higher rates of disability, cognitive impairment, and all-cause mortality, particularly in rural areas (57). Considering the importance of access to healthcare for healthy aging, our global community struggles to provide appropriate and timely access to healthcare for people aged 65 or older (58). Our difficulty to provide relevant and effective services for older adults is partially attributed to rapidly shifting demographics. As the number of projected older adults grows from about 524 million in 2010 to nearly 1.5 billion in 2050, the projected number of younger people (ages 0–25 years) is expected to decrease (59). This phenomenon shifts the dependency ratio and results in an increasing percentage of older adults in the global population (see Figure 2).

Still more of our difficulty providing access to care may be explained by shifts in disease burden. As our global population steadily ages, the chronic diseases highly associated with aging (e.g., heart disease, stroke, and COPD) now pose the greatest threat to global health (59). In 2000, dementia was the 14th leading cause of death worldwide, but by 2016, dementia-related deaths rose to the 5th leading cause of death (60). Across the globe, healthcare systems may be ill-prepared to shift resources to meet the demand for geriatric care and chronic disease treatment and management. Table 3 provides an overview of selected evidence documenting the relationship between access to care and healthy aging from different countries.

**Table 3:** Example studies documenting the relationship between access to care and healthy aging.

**References**	**Narrative Summary**	**Country**
Ploughman et al. (51)	Older adults with multiple sclerosis in Canada report “effective and accessible” healthcare as a key factor in healthy aging	Canada
Heller and Sorenson (56)	Access to proper screenings and preventive services for people with intellectual disabilities in the United States, facilitate healthy aging	United States
McWilliams (52)	There are significant benefits of coverage for adults with acute or chronic conditions for which there are effective treatments	United States
Van Der Wees et al. (53)	Within low-income households, Massachusetts healthcare reform was associated with and greater use of certain preventive services, relative to other New England states	United States
Sommers et al. (54)	Association between Health reform in Massachusetts and significant reduction in all-cause mortality and mortality from causes preventable by healthcare	United States
Courtemanche and Zapata (55)	Healthcare reform in Massachusetts improved overall self-assessed health. These health effects were strongest among non-whites, near-elderly adults, women, and individuals with low incomes	United States
Okoro et. al (42)	Older adults more likely to receive preventive services, with access to sources of regular healthcare	United States
Zhang et al. (57)	Significant association between inadequate healthcare among older adults and higher rates of disability, cognitive impairment and all-cause mortality, particularly in rural areas	China

## Improving Access to Healthcare Among Older Adults

A lack of financial resources leads to poor health, which can, in turn, lead to a dangerous cycle of further impoverishment (19). Future disparities in mortality based on income inequalities in older adults could diminish with the implementation of interventions designed to reduce barriers to care among younger populations (61). Internationally, interventions that either directly improve healthcare access (e.g., by expanding health insurance) or focus on alleviating financial disparities typically lead to improved health among older adults (62–64).

Mexico experimented with income supplementation for older adults. Elderly residents of two states in the Yucatan who received income supplementation (i.e., a 44% increase in household income) spent their extra income on doctor visits and medications, and realized improved health outcomes (62). In Italy, socioeconomic disparities in influenza vaccinations exist among adults, but not among older adults. Influenza vaccines are free for older adults, thus potentially remediating any socioeconomic effect in vaccine uptake (63). In the United States, expanding state Medicaid (public health insurance) programs resulted in decreased rates of uninsured older adults, reduced forgoing care due to costs, and a significant decrease in mortality among older adults living in expansion states (64) Increasing health insurance coverage also resulted in improved access to care in China (65). Table 4 provides an overview of selected evidence documenting strategies to improve access to healthcare among older adults from different countries.

**Table 4:** Example studies documenting strategies to improve access to healthcare among older adults.

**References**	**Narrative Summary**	**Country**
Aguila (62)	Income supplementation for older adults	Mexico
Damiani et al. (63)	Influenza vaccines are free for older adults	Italy
Sommers et al. (64)	Expanding state Medicaid (public health insurance) programs resulted in decreased rates of uninsured older adults, reduced forgoing care due to costs, and a significant decrease in mortality among older adults living in expansion states	United States
Zhang et al. (65)	Increasing health insurance coverage also resulted in improved access to care in China	China

## Discussion

The rapid growth of the global aging population alongside projected decreases in younger demographics (59) will pose new and intensified access barriers and burdens on healthcare systems worldwide. This scenario highlights the importance of building and supporting healthcare infrastructure and processes to effectively identify the healthcare needs of older adults and efficiently serve them with quality services. With a growing consumer base and unchanged healthcare systems, our ability to adequately care for an aging society will become labored and severely compromised, which will diminish health outcomes and opportunities for healthy aging.

As documented in this mini-review, substantial evidence exists to support the strong interplay between socioeconomic status (SES), healthcare access, and healthy aging. Several studies document the relationship between SES, healthcare utilization, and health outcomes among older adults across the globe. Universally, the majority of studies show that lower SES is associated with more access barriers (10–16), which is subsequently associated with worse health outcomes and premature death. Compounding disparities exist which exacerbate these relationships among people of color and other minority or traditionally disenfranchised groups (36, 65). Evidence suggests that removing the financial barriers to healthcare access such as providing universal healthcare coverage in European countries or Medicare in the United States (64) can improve health outcomes.

Traditionally, the Anderson Behavioral model contextualizes healthcare access as a function of predisposing, enabling, and need-related factors (66). While this is a strong approach to understand the factors associated with healthcare access, it is an individual-level model and requires additional context to apply to larger communities and populations. The issue of healthcare access among older adults has upstream and downstream elements and considerations. Because aging begins at birth, the outcomes that manifest in older adulthood have origins in earlier years that are more formative. The SES of an individual throughout their life-course can enhance or suppress disease and other healthcare needs. And, patterns of healthcare utilization in earlier years can characterize older adult healthcare access and utilization patterns.

### Recommendations for Future Research

Surveillance efforts and research are needed to better understand the trends in aging and associated disparities in SES and healthcare access worldwide, especially in light of the seemingly paradoxical findings in the recent literature. However, measurement issues complicate our ability to fully understand the relationship between SES and healthcare access among older adults. In the field of health services research, SES is not uniformly measured, which has vast implications for advancing healthy aging. Measurement of SES in health services research is difficult, due to the variety of definitions and constructs measured under this concept (67–69). Choice of definition and measurement affects the outcomes of disparity research. Considering the complexity of appropriately measuring SES (a combination of education, employment, and income), multidimensional SES measures (i.e., household income vs. community wealth) may create a more accurate picture of the drivers of health disparities (70, 71). Unfortunately, many studies simply measure and utilize one aspect of SES (e.g., household income), which does not fully encapsulate SES and only serves as a proxy to the theorized construct. Or, studies include multiple measures of SES, but include them in statistical models as separate variables, not accounting for multicollinearity and interdependence. This measurement issue is further complicated when measuring SES in later life because of the complexities of SES based on educational norms of decades past, years post-retirement, and subsidized healthcare in advanced age. As such, it is suggested that SES is better measured by wealth instead of income as people age and retire (72). This ultimately has practical implications that influence the associated findings, interpretations, and recommendations for action to improve healthcare access within the field. For example, while increasing health insurance coverage resulted in improved access to care in China, income-related disparities still existed. Higher-income older adults accessed more outpatient services (65). This suggests a need to further explore the relationship between income, health insurance, and healthcare access among older adults. Developing standardized and evidence-based guidelines for trend surveillance among these factors would allow more accurate and consistent comparisons between countries and other contexts. Likewise, creating expert consensus on measurement issues related to SES is necessary to improve our understanding of the relationship between SES, access to care, and healthy aging, and facilitate moving beyond mortality as the major outcome of that relationship.

Furthermore, we find diminishing returns as marginalized populations attain higher socioeconomic statuses, with people of color experiencing less health benefits than whites from socioeconomic attainment (73). In a study of preventative screenings among women living in the United States, Monnat found that low SES women were less likely to receive a mammogram or pap smear (74). As SES increased, white women were more likely than women of color to receive these services. In what Monnat referred to as “paradoxical returns,” the likelihood that Asian women received a mammogram or Pap smear decreased as SES increased. Even when healthcare services are introduced in a community by reducing the proximity to clinics or reducing socioeconomic barriers, there is no guarantee that target populations, including older adults, will access and utilize these services. Moving forward, we must consider the upstream and downstream effects of SES on healthcare access. While SES hinders the ability of older adults to access healthcare services, improving their financial situation in any capacity alleviates burdens and stressors that can improve their healthcare use patterns and health outcomes. For example, linking older adults to services that save them money (e.g., congregate meal programs, medication assistance programs, transportation services) increases the chances they will use the unused funds for needed healthcare.

Opportunities exist to rethink traditional healthcare to provide complementary services that are free or low-cost. While healthcare is often considered to take place within clinical settings, other resources, services, and programs can assist older adults in the community. For example, in the United States, the federal government has supported a number of evidence-based programs for low-income and vulnerable older adults to help them improve health outcomes (e.g., fall prevention, chronic disease self-management) (75–77). These programs transcend traditional clinical settings and complement healthcare while increasing access to health services.

There is a projected increase in the number of older adults, and an attendant decrease in the number of younger adults in the years to come (59). This projection brings to light the importance of putting structures in place that identify the needs and challenges of providing care to older adults, as well as an estimation of the resources needed to ensure older adults have healthcare access to improve their health outcomes. Properly assessing SES among older adults is essential for providing older adults with basic and necessary healthcare access and services. Although programs like Medicaid in the United States have helped in this regard, more can be done (64). At current, the healthcare system is ill-equipped to handle the volume of older adults requiring healthcare, which creates an inverse supply-demand ratio. In short, even if access to healthcare is improved for those with low SES, the healthcare system may be unable to adequately serve the influx in older adult patients. However, because evidence supports that financial resources are directly proportional to good health (19), efforts are needed to support individuals and the healthcare system with financial resources. This can be most effective with grassroots approaches and interventions to improve SES among older adults and through data-driven policy and systems change.

## Caveats

While access to care is necessary for health aging, focusing on access alone is not sufficient to improve the health of populations (78). Access to health care can improve health outcomes, but most likely only to a certain degree, necessitating a complementary focus on social determinants (79). In some instances, more healthcare doesn't equal “more” health. In Taiwan, increased access to health care through health insurance and the resulting increase in health services utilization did not affect mortality or self-perceived health (80). Similarly, more expensive healthcare isn't linked to better outcomes, at least among Medicare recipients in the United States (81). In Brazil, where older adults have access to healthcare through a public health care system and private insurance, patient perceptions affect treatment initiation and glycemic control of Type II diabetes (82). Improving access to healthcare by reducing financial disparities and improving wealth among older adults is a vital first step but should not be seen as the final step in ensuring global healthy aging.

## Author Contributions

DM, OO, and MS conceptualized, wrote, and reviewed the manuscript.

## Conflict of Interest

The authors declare that the research was conducted in the absence of any commercial or financial relationships that could be construed as a potential conflict of interest.
